# Osmotic and diffusive flows in single-file pores: new approach to modeling pore occupancy states

**DOI:** 10.1186/s12976-018-0087-8

**Published:** 2018-10-01

**Authors:** Gordon Kepner

**Affiliations:** Membrane Studies Project, P.O. Box 13160, Minneapolis, MN USA

**Keywords:** New combinatorial analysis, Tracer translocation, Knock-on collisions, Brownian fluctuations, Vacancy translocation, Tracer exit states

## Abstract

**Background:**

The relation between osmotic permeability, P_f_, diffusion permeability, P_d_, and the number of water molecules, N_p_, in the single-file membrane pore remains an open question. Theoretical analyses, empirical studies on aquaporins and nanotubes, and molecular dynamics simulations have yet to provide a consensus view.

**Results:**

This paper presents a new combinatorial analysis of the different pore states formed from water molecules and the presence of a vacancy that differs from the several previous combinatorial approaches to analyzing pore states. It is the first such analysis to show that P_f_ / P_d_ = N_p_. It is rooted in the concept of different classes of pore occupancy states, tracer states and tracer exit states, present in the pore. This includes pores with and without a single vacancy. The concepts of knock-on collisions and concerted Brownian fluctuations provide the mechanisms underlying the behaviors of the tracer and vacancy as each moves through the pore during osmotic or diffusive flow. It develops the important role of the knock-on collision mechanism for osmotic flow. An essential feature of the model is the presence, or absence, of a single vacancy in the pore. The vacancy slows down tracer translocation through the pore. Its absence facilitates osmotic flow.

**Conclusions:**

The full pore states and the single vacancy states together with the knock-on and Brownian mechanisms account for the relative values of P_f_ and P_d_ during osmotic and diffusive flow through the single-file pore. The new approach to combinatorial analysis differs from previous approaches and is the first to show a simple intuitive basis for the relation P_f_ / P_d_ = N_p_. This resolves a long persisting dichotomy.

## Background

The relationship between osmotic permeability (P_f_) and diffusion permeability (P_d_) for water movement across cell membranes in the single-file pore case was intensively studied over the last half of the 20th century [1–3]. The idea that experimentally observed differences in P_f_ and P_d_ revealed the existence of pores for water permeation in membranes was first put forth based on experimental studies in frog skin [4]. Multiple studies using the combinatorial analysis of pore occupancy states, based on varying analyses of the stochastic nature of this movement through pores, had concluded that flux ratios depended on (N_p_ + 1), [1, 5–7].

The 21st century has seen renewed interest in this issue because of the availability of artificial nanotubes and biological aquaporins [3, 8–11]. Recent theoretical analyses using Molecular Dynamics, as well as experimental studies, have employed nanochannels as models with some of the physical properties of biological protein water channels (aquaporins), see reviews [3, 9, 11, 12]. Parisi et al. [3] ask, “Which is the molecular mechanism that differentiates diffusion from osmosis in a “single-file” channel?”

The exact dependence of P_f_ / P_d_ on N_p_ continues to generate divergent views, generally as a result of different assumptions about the stochastic mechanisms involved in water permeation through these single-file pores. This continues to result in differing approaches to the combinatorial analysis of the pore occupancy states present. None agreed with Levitt’s [13] result reached via a non-combinatorial analysis approach, giving P_f_ / P_d_ = N_p_. These conflicting results from multiple studies in the literature invite a renewed effort to resolve this persistent dichotomy among the various papers employing the combinatorial analysis of presumed pore states.

In order to resolve this, a new model is introduced here with modified assumptions about the nature of pore states, particularly the role of the single vacancy. This involves distinct classes of pore states — tracer states and tracer exit states — that arise in the pore, including pores with and without a single vacancy. It leads to a different combinatorial analysis model that is the first to show P_f_ / P_d_ = N_p_.

## Methods — Theoretical Approach

The aim of the study is to develop a new perspective on the concept of pore states that will yield a better analysis of the problem relating P_f_ / P_d_ to pore occupancy. Whether the ratio P_f_ / P_d_ for a single-file pore is found to be N_p_, (N_p_ + 1), N_p_ – 1, or other depends on the model used to derive P_f_ and P_d_ and is significantly affected by the assumption of either one or no vacancy allowed in the pore [1, 5, 8, 10, 14, 15]. For the full pore experiencing osmotic flow, it is assumed in some studies that all the water molecules in the pore move together at the same time to shift a molecule at the entrance slot of the pore to the exit slot and then out of the pore into solution [1, 15–19]. The single-file pore model has also been investigated extensively in studies of ion permeation in channels such as the K^+^ channel [18, 19], giving results not directly comparable to permeation by an electrically neutral water molecule through an uncharged water pore.

Assume an initially full single-file pore of water molecules. The pore is divided into slots of length equal to the diameter of the water molecule. On both the left-hand-side (pore entrance slot) and the right-hand-side (pore exit slot), there is an exterior solution.

Assume three water molecules fill this pore, denoted by OOO. The model considers the roles of two physical mechanisms capable of causing a water molecule in the exit (or entrance) slot to leave the pore — thereby creating a vacancy at an end slot.

One is a “knock-on” billiard-ball, inelastic collision between a water molecule from the solution adjacent to the occupied entrance slot and the water molecule in the entrance slot of the pore. This transfers momentum through the water molecules in the full pore to the molecule in the exit slot, causing it to leave the pore. If the two solutions are in equilibrium, the collision frequency, on average, should be the same at each end of the pore. If not, the solution at the pore’s entrance slot, with higher chemical activity of water relative to the exterior solution at the exit slot, should on average produce a greater frequency of collisions at the pore entrance slot. The other is Brownian fluctuations that, over some time interval, could randomly produce more “jumps” to the left, or right, causing a water molecule at either end slot to leave the pore. This is particularly sensitive to the presence of a vacancy in the pore.

Each mechanism can create a vacant slot, denoted by [], at either end of the pore, OO[] or []OO. This vacant slot could then be filled in one of two ways:

- A water molecule inside the pore, the middle one in the example with three slots, jumps into the vacant exit or entrance end slot, giving O[]O.
- A water molecule from the exterior solution jumps into the vacant exit or entrance end slot, which reestablishes the full pore, OOO.

It is usually assumed that the activation energy for (a) is less than for (b), which leads more often to O[]O. The energetics of water movement at the entrance and exit slots are crucial events for the mechanisms of diffusive and osmotic flow through the single-file pore, Davis et al. [12].

Experimental observations with tracers show that water molecules can diffuse through the pore equally from one side to the other under equilibrium conditions. Assume a vacancy is created at the exit slot OO[] (right-hand side) and the tracer, denoted by **O**, is located in the exterior solution adjacent to the pore entrance (left-hand-side). The Brownian fluctuations in the molecules inside the pore favor jumping into any available adjacent vacancy in the pore. Thus, this mechanism acts on the two remaining molecules in the pore and tends to move them toward the exit slot vacancy. Therefore, the vacancy then moves toward the entrance slot, eventually giving []OO. Now a tracer water molecule, **O**, can jump into this vacancy at the entrance slot.

**O** OO[]

**O** O[]O

**O** []OO

**O**OO

What mechanism (under equilibrium conditions) causes it to be transported through the length of the pore to the exterior solution adjacent to the exit end of the pore, going from **O**OO ––> OO**O**, then exiting the pore to create OO[]?

Over some short time interval this tracer could occasionally experience three more rightward random Brownian jumps than leftward ones. This could allow it to escape the pore. The longer the pore (with N_p_ water molecules), the lower the chances of experiencing the required N_p_ extra rightward jumps in this short time interval and the longer it takes to escape the pore. This process works equally well in either direction through the pore. Effectively, it acts to slow down the movement of the tracer through the pore.

This introduces an additional mechanism that produces net movement of water molecules through the pore. Assume an impermeable solute placed in the solution exterior to the pore exit. There is a net increased collision frequency, due to the water molecules of the exterior solution (pure water), at the entrance slot of the pore. This produces two effects:

- When the pore is full, a collision at the entrance slot transfers momentum through the pore’s water molecules to the water molecule in the exit slot, which causes it to leave the pore and create a vacancy at the exit slot, as shown below.

Collision O ––> **O**OO ––> **O**O[] + O

b) When there is a vacancy created at the exit slot, this net increase in collision frequency at the entrance slot continues to produce additional repetitive collisions at the pore entrance, see below.

Collision O ––> **O**O[] ––> **O**[]O + O

The middle water molecule then moves into the exit slot vacancy. This is more likely than having a water molecule from the exterior solution jump into the vacant exit slot. The next collision with the water molecule occupying the entrance slot moves it into the vacancy in the middle slot.

Collision O ––> **O**[]O ––> []**O**O

A water molecule can then jump into this newly vacant entrance slot from the exterior solution.

Collision O ––> []**O**O ––> O**O**O

This increased repetitive collision mechanism at the pore entrance favors maintaining a full pore, which is a requirement for the collision mechanism to move a molecule out of the exit slot into the exterior solution.

On average, because of the osmotic gradient, this sequence occurs more often than a similar sequence of collisions from the exterior solution at the exit slot, leading to net osmotic water flow through the pore from the entrance to the exit slot. The sequence repeats until the tracer reaches the exit slot and then is moved out of the pore into the exterior solution by a collision. Thus, the vacancy moves more rapidly than in the diffusion case from the exit slot to the entrance slot, where it can then fill from the exterior solution. Effectively, this reduces the lifetime (T_v_)_osm_ of the vacancy in the pore (how long it exists there on average before replaced by a water molecule) so that during osmotic flow the value of this (T_v_)_osm_ becomes very much shorter than the lifetime (T_v_)_diff_ of any vacancy occurring in the pore during diffusion, which increases osmotic flow through the single-file pore relative to diffusive flow. The relative imbalance between the collision and Brownian mechanisms is a function of pore length (N_p_). It is less for shorter pores. It disappears, as shown in experiments on lipid bilayers having no pores [2].

## Results and discussion

### Model

The new combinatorial analysis approach developed here assumes:

- There are *i* slots of diameter equal to the diameter of a water molecule, d_w_, where *i* ≥ 1. The pore’s length is then *i* · d_w_ = *L*_pore_. Thus, *i* = N_p_ when the pore is full of water molecules.
- There are three different objects that can occupy a slot in the pore. These are the physically indistinguishable water molecules O, the water tracer molecule **O**, and the vacant slot [].
- No more than one **O** or one [] is allowed in the pore at any time.

Using basic combinatorial principles, let (*N*_S_)_*i*_ be the total number of pore states for the three objects for a given number of slots, *i*, in the pore. The ordering of the objects in the pore also matters. Then calculate (*N*_S_)_*i*_ as a function of *i* to obtain P_f_ and P_d_ in terms of the relevant pore states, the number of water molecules and the presence or not of a vacancy in the pore. There are four cases to consider, all mutually exclusive.

1. No **O** or [] in the pore, only O, giving one such pore state.
2. One **O**, no [] and the rest O, giving *i* pore states that could have one **O**, no [], and only one way to fill the remaining slots with O, which therefore yields *i* pore states.
3. One [], no **O**, and the rest O, which yields *i* pore states.
4. One **O** and one [], with the remaining slots filled with O. Now select a slot for the [] from the N_sl_ slots present in the pore. There are *i* ways to do this. Now choose one of those [*i* – 1] remaining slots for **O**. There are then *i* · [*i* – 1] ways to arrange the **O** and the [] in the pore, but only one way to fill the remaining slots with O. The total number of pore states in this case equals, *i* · [*i* – 1] = *i*^2^ – *i*.

The total number of pore states is (*N*_S_)_*i*_ = *i* · [*i* – 1] + *i* + *i* + 1 = *i*^2^ + *i* + 1. For the detailed example used below, *i* = 3, so (*N*_S_)_3_ = 13 unique pore states. At the limit of *i* = 1, this gives (*N*_S_)_1_ = 3 pore states.

### Pore occupancy states

The concept of pore states seeks to describe the occupancy configurations of the single-file pore. Thus, the mechanisms for moving a water molecule out of the pore that are considered here —knock-on collision and concerted Brownian fluctuations — can only act on the occupancy states actually present. Exiting a pore requires the water molecule to be in the end slot of a pore state configuration. The model used here revisits the concept of pore states [11, 14]. It introduces new classes of these states and relates them to P_f_ and P_d_ to obtain P_f_ / P_d_. Table 1 presents these different pore states for *i* = 3, using the combinatorial model with (*N*_S_)_*i*_ = *i*^2^ + *i* + 1 = 13.

**Table 1:** Four different types of pore occupancy states, for *i* = 3

Pore Occupancy	Pore States *i* = 3	Diffusion Tracer States	Diffusion Tracer Exit States	Osmotic Tracer States	Osmotic Tracer Exit States
	*N*_S_(*i)*	*N*_S_(*i*)_d_	*N*_S_(*i*)_dex_	*N*_S_(*i*)_f_	*N*_S_(*i*)_fex_
OOO	1				
[]OO O[]O OO[]	3				
**O**OO O**O**O OO**O**	3	3	1	3	1
**O**O[] O**O**[] **O**[]O O[]**O** []**O**O []O**O**	6	6	1 1		
Total States	13 *i*^2^ + *i* + 1	9 *i* ^2^	3 *i*	3 *i*	1

This specifies quantitatively the four types of tracer states (one tracer in the pore) that can exist within a pore with one vacancy and within a filled pore as well.

1. Diffusion Tracer States (*N*_S_)_d_ that contain the tracer **O**, giving nine for *i* = 3, as shown in Table 1.
2. Diffusion Tracer Exit States (*N*_S_)_dex_ that contain a tracer **O** in the exit slot of the pore, giving three for *i* = 3.
3. Osmotic Tracer States (*N*_S_)_f_ with no vacancy and with a tracer **O**, giving three for *i = 3.*
4. Osmotic Tracer Exit States (*N*_S_)_fex_ with no vacancy and with a tracer **O** in the exit slot, giving one for *i* = 3.

This differs from “pore state,” which can include states without a tracer [8, 14, 15, 17]. It also defines the new concept of tracer exit states for osmotic flow, (*N*_S_)_dex_, and diffusion flow, (*N*_S_)_fex_. These include only pore configurations with the tracer in the exit slot of the pore.

Osmotic flow is dependent on the three osmotic tracer states (*N*_S_)_f_ — full pore states. If the activity of water is highest at the pore entrance, then net osmotic flow occurs out of the exit slot. When there is a vacancy, this increased collision frequency at the pore entrance acts to rapidly fill vacancies arising at the pore entrance and exit slots, favoring the full pore state.

For diffusive flow, there are three states with a tracer in the entrance slot and three with a tracer in the exit slot, see Table 1 and (*N*_S_)_d_. Under the equilibrium condition, there can be no net flow. Yet, a tracer can make its way through the pore in either direction, equally, via the Brownian fluctuation mechanism. Similarly, the collision mechanism acts equally at both ends of the pore in promoting escape from the pore.

The sequential translocation of tracer and vacancy moves the tracer toward the exit and the vacancy toward the entrance to the pore. Six pore occupancy states contain a vacancy and a tracer. This vacancy is more likely to be filled from within the pore because it is usually assumed that the energy barrier for exiting or entering the full pore is significantly greater than for moving into a vacancy within the pore [1, 8]. The relative energy barrier is less for a molecule moving from the exterior solution into the vacancy at the pore entrance site. This causes the tracer to remain longer within the pore and inhibits exiting. Only the three full pore occupancy states (*N*_S_)_f_, Table 1, with a tracer create the full pore condition favoring exit from the pore via the knock-on mechanism, which operates equally on these states.

The tracer measures one-way translocation via diffusion within the pore, under equilibrium conditions. It is the same in both directions. The effect of the vacancy is to decrease greatly the rate at which a tracer can diffuse through the pore. When the vacancy reaches the entrance slot, it may fill from the exterior solution with either a tracer or non-tracer. If instead it fills from within the pore, this creates a vacancy at the exit slot, for the *i* = 3 case. This process repeats again and again as long as there is one vacancy in the pore. Thus, what matters is the number of tracer exit states available as pathways through the membrane for osmotic flow (*N*_S_)_fex_ and diffusion (*N*_S_)_dex_. In the limit *i* = 1, there are just 3 pore occupancy states. Therefore, the total states row in a table for *i* = 1 would take the values: 3, 1, 1, 1, 1.

### Tracer states — (*N*_S_)_d_ and (*N*_S_)_f_

Table 1 shows the 9 diffusion tracer states. Thus, (*N*_S_)_d_ = *i* + *i* · (*i* – 1) = *i*^2^ = 9. For osmotic flow, (*N*_S_)_f_, there are *i* = 3 states. Assume as with Finkelstein [1] that the P_f_ / P_d_ = [(1 / (N_S_)_f_] / [1 / (N_S_)_d_], while recognizing that (N_S_)_f_ = *i* and (N_S_)_d_ = *i*^2^, so (1/*i*) / (1/*i*^2^) = *i* or simply (1 / L) / (1 / L^2^) = L. The nominal dependence of (N_S_)_f_ on (1 / L) is effectively cancelled out in this relation. Therefore,

$$ {P}_f/{P}_d=\left(1/{N}_{\mathrm{S}}\right)\mathrm{f}/\left(1/{N}_{\mathrm{S}}\right)\mathrm{d}=\left(1/3\right)/\left(1/9\right)=3=i=\mathrm{Np}=3\ \mathrm{water}\ \mathrm{molecules}\ \mathrm{in}\ \mathrm{pore} $$

Defining P_d_ in terms of pore states depends on whether the pore is always full or has one vacancy. If full, there are 3 tracer states and 1 null state (first column, Table 1), which equals (*i* + 1). If there is one vacancy, then there are *i*^2^ states, only 6 of which are tracer states. For the full and vacancy states there are (*i*^2^ + *i* + 1) = 13 pore states.

### Tracer exit states — (*N*_S_)_dex_ and (*N*_S_)_fex_

Another approach emerges from considering *only* the tracer *exit* states, (*N*_S_)_dex_ and (*N*_S_)_fex_, shown in Table 1.

$$ \frac{{\mathrm{P}}_{\mathrm{f}}}{{\mathrm{P}}_{\mathrm{d}}}=\frac{1/{\left({N}_{\mathrm{S}}\right)}_{\mathrm{f}\mathrm{ex}}}{1/{\left({N}_{\mathrm{S}}\right)}_{\mathrm{d}\mathrm{ex}}}=\frac{1/1}{1/3}=3=i={\mathrm{N}}_{\mathrm{p}}=3\ \mathrm{water}\ \mathrm{molecules}\ \mathrm{in}\ \mathrm{pore} $$

Thus, as the pore lengthens, (*N*_S_)_dex_ = *i* steadily increases, while (*N*_S_)_fex_ stays constant.

The essential events for moving water into and out of the pore necessarily occur at the entrance and exit slots. The new Tracer Exit States approach surprisingly suggests that P_f_ does not depend on *i* and therefore not on pore length, because (*N*_S_)_fex_ will always have a value of 1, regardless of *i* and therefore of pore length. Theoretical studies suggest overall conductance is weakly dependent on pore length [12, 14]. This result is supported by theoretical simulation studies on a ‘greasy’ or frictionless nanopore [16, 20], see also [8]. Saparov’s experimental study [21] showed P_f_ did depend exponentially on pore length; but it also pointed out that “If full occupancy is assumed or if the number of vacancies is limited to one (the cases analyzed here), the effect *of N* on *p*_*f*_ has to be much weaker.” This appears puzzling but is consistent with recognizing that (N_S_)_dex_ / (N_S_)_fex_ depends primarily on (N_S_)_dex_ and this certainly increases with increased pore length. There is no study that has independently measured P_f_ / P_d_ and the single-file pore length in order to test whether P_f_ / P_d_ = N_p_, see also Table 2 below.

**Table 2:** Theoretical expressions for P_f_ / P_d_ with N_p_ = *i*

Actual *i* = N_p_	(*N*_S_)_d_ / (*N*_S_)_f_ = (*N*_S_)_dex_ / (*N*_S_)_fex_ = *i*	*i* – 1	(*i*^2^ + 1) / (*i* + 1)	*i* + 1
1	1	0	1	2
2	2	1	1.67	3
3	3	2	2.5	4
4	4	3	3.4	5
7	7	6	6.25	8
10	10	9	9.2	11

This single vacancy model is compatible with the “chain-like” concept of water movement within the single-file pore suggested by some molecular dynamics studies. The vacancy could lead to brief coordinated fluctuations arising to move several water molecules in concert toward the vacancy. This more rapidly shifts the vacancy, by several slots, in the opposite direction.

### Comparing P_f_ / P_d_ analyses

Table 2 summarizes some of the differing results for P_f_ / P_d_ taken from the literature cited here [1, 5–8, 10, 14, 15].

**Fig. 1:**
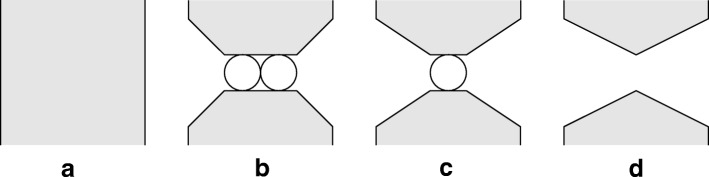
Limiting cases pore length (**a**) the lipid bilayer membrane with no pores, (**b**) pore with *i* = 2, (**c**) pore with *i* = 1, (**d**) membrane with just a ‘hole’, not to scale

The similarity of the results at larger *i* (actual biological aquaporins) would make it quite difficult to distinguish among them on an experimental basis. The cases that offer the greatest relative differences among these theoretical expressions for P_f_ / P_d_ are the limiting cases, as shown in Fig. 1.

For (**a**) it is well established both theoretically and experimentally that P_f_ / P_d_ = 1, [2]. For (**b**), only (*N*_S_)_d_ / (*N*_S_)_f_ = (*N*_S_)_dex_ / (*N*_S_)_fex_ gives 2. For (**c**) with *i* = 1, then (*i* − 1) = 0, and it is difficult to understand why P_f_ / P_d_ = (*i* + 1) = 2. For (**d**), there is no pore wall to interact with and constrain the movement of the water molecules. These limiting cases of very short pores are inaccessible experimentally. They might be investigated using simulation analyses.

As shown in Table 2, when *i* > 1, then [*i*^2^ + 1] / (*i* + 1) yields non-integer values, which are difficult to interpret unless rounded to the nearest integer. As *i* becomes large, then the relation (*i*^2^ + 1) / (*i* + 1) does approach *i* more closely. The following relationship is supported by some models [10]: P_f_ / P_d_ = < *n* > + 1, where now < *n* > is the average occupancy of the pore containing *i =* N_p_ molecules, when all slots are full. The model developed here allows for one vacancy in the pore. This suggests that < *n* > = *i* – 1, giving, P_f_ / P_d_ = (*i* – 1) + 1 = *i*. Only the tracer states model presented here was able to produce the relationship P_f_ / P_d_ = *i* = N_p_, the number of water molecules in the full pore. This result based on the new combinatorial analysis of pore states is also supported by Levitt’s [13] different theoretical approach to this problem.

## Conclusions

The new approach to combinatorial analysis of pore states differs from previous approaches and is the first to show a simple intuitive basis for the relation P_f_ / P_d_ = N_p_. This resolves the dichotomy that has so long persisted. The model developed here does not depend on a physical description of the interactions occurring within the pore among the water molecules, or with the pore wall. One essential feature is the presence of the vacancy, which forces the tracer molecule to spend more time “sampling” each of the possible tracer states — thereby significantly slowing its passage through the pore and so increasing P_d_. Another is the introduction of a revised concept of pore states along with a clear distinction between tracer and tracer exit states, which relates directly to P_f_ / P_d_.

These results provide a way to reconcile the various models used to analyze the important ratio P_f_ / P_d_. It offers a unifying intuitive explanation in terms of pore occupancy states (based on tracer and tracer exit states) for the osmotic and diffusive flows single-file pore. The roles of the full pore states and the single vacancy states are shown to be coherent with the mechanisms postulated for diffusive and osmotic flows in the single file pore. This suggests new insights in terms of the concepts of tracer and exit states that simplify the understanding of osmotic and diffusive flows through such pores.
